# Diethyl 2,6-dimethyl-4-phenyl-1,4-dihydro­pyridine-3,5-dicarboxyl­ate

**DOI:** 10.1107/S1600536809009118

**Published:** 2009-03-19

**Authors:** Ming-Sheng Bai, Yan-Yun Chen, Dong-Ling Niu, Li Peng

**Affiliations:** a College of Life Science, Ningxia University, Yinchuan 750021, People’s Republic of China

## Abstract

The title mol­ecule, C_19_H_23_NO_4_, was synthesized by the reaction of benzaldehyde, ethyl acetoacetate and NH_4_HCO_3_. The dihydro­pyridine ring adopts a flattened boat conformation and the plane of the base of the boat forms a dihedral angle of 88.78 (9)° with the phenyl ring. The packing is stabilized by strong inter­molecular N—H⋯O and weak inter­molecular C—H⋯O hydrogen bonds.

## Related literature

For general background, see: Cutshall *et al.* (2002 ▶); Henry (2004 ▶). For the crystal structure of the related compound diethyl 2,6-dimethyl-4-styryl-1,4-dihydro­pyridine-3,5-dicarb­oxyl­ate, see: Wang *et al.*, (2007 ▶). For hydrogen bond definitions, see: Desiraju & Steiner (1999 ▶).
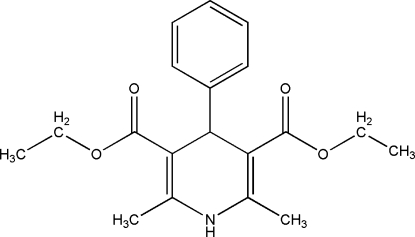


## Experimental

### 

#### Crystal data

C_19_H_23_NO_4_
*M* *_r_* = 329.38
Monoclinic, 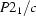

*a* = 9.7502 (12) Å
*b* = 7.3854 (9) Å
*c* = 24.326 (2) Å
β = 92.567 (1)°
*V* = 1749.9 (3) Å^3^
*Z* = 4
Mo *K*α radiation
μ = 0.09 mm^−1^
*T* = 298 K
0.50 × 0.46 × 0.32 mm

#### Data collection

Siemens SMART 1000 CCD diffractometer
Absorption correction: multi-scan (*SADABS*; Sheldrick, 1996 ▶) *T*_min_ = 0.958, *T*_max_ = 0.973
8718 measured reflections
3084 independent reflections
1989 reflections with *I* > 2σ(*I*)
*R*_int_ = 0.033

#### Refinement

*R*[*F* ^2^ > 2σ(*F* ^2^)] = 0.045
*wR*(*F* ^2^) = 0.131
*S* = 1.01
3084 reflections
225 parameters
H atoms treated by a mixture of independent and constrained refinement
Δρ_max_ = 0.22 e Å^−3^
Δρ_min_ = −0.18 e Å^−3^

### 

Data collection: *SMART* (Siemens, 1996 ▶); cell refinement: *SAINT* (Siemens, 1996 ▶); data reduction: *SAINT*; program(s) used to solve structure: *SHELXS97* (Sheldrick, 2008 ▶); program(s) used to refine structure: *SHELXL97* (Sheldrick, 2008 ▶); molecular graphics: *SHELXTL* (Sheldrick, 2008 ▶); software used to prepare material for publication: *SHELXTL*.

## Supplementary Material

Crystal structure: contains datablocks I, global. DOI: 10.1107/S1600536809009118/fb2141sup1.cif

Structure factors: contains datablocks I. DOI: 10.1107/S1600536809009118/fb2141Isup2.hkl

Additional supplementary materials:  crystallographic information; 3D view; checkCIF report

## Figures and Tables

**Table 1:** Hydrogen-bond geometry (Å, °)

*D*—H⋯*A*	*D*—H	H⋯*A*	*D*⋯*A*	*D*—H⋯*A*
N1—H1⋯O4^i^	0.81 (3)	2.19 (3)	2.986 (3)	168 (3)
C3—H3⋯O1	0.98	2.35	2.733 (3)	103
C3—H3⋯O4	0.98	2.43	2.816 (3)	103
C7—H7⋯O1	0.93	2.55	3.169 (3)	124
C12—H12*C*⋯O2	0.96	2.27	2.841 (3)	116
C15—H15*A*⋯O3	0.96	2.42	2.762 (3)	101
C8—H8⋯O2^ii^	0.93	2.51	3.387 (3)	157

Symmetry codes: (i) 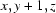; (ii) 
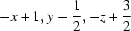
.

**Fig. 1.**
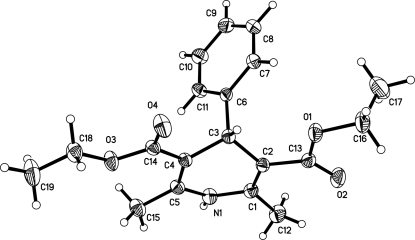
The title molecule with the atomic numbering scheme. The displacement ellipsoids are shown at the 30% probability level.

## supplementary crystallographic information

### Comment

The development of new methods for the synthesis of substituted pyridines is a motive for the current study. Substituted pyridines attract the interest because of their presence in numerous natural products along with a wide spectrum of their physiological activities (Cutshall *et al.*, 2002). Pyridine derivatives and their complexes have been studied for their fungicidal and antibacterial effects, as well as antiviral drugs (Henry, 2004).

In this paper, we present the structure of diethyl 2,6-dimethyl-4-phenyl-1,4-dihydropyridine-3,5-dicarboxylate (Fig. 1).

The bond lengths and angles are normal and comparable to those observed in the reported diethyl 2,6-dimethyl-4-styryl-1,4-dihydropyridine-3,5-dicarboxylate (Wang *et al.*, 2007).

In the crystal structure, the dihydropyridine ring adopts a flattened boat conformation and the plane of the base of the boat (C1/C2/C4/C5) contains 88.78 (9)° with the phenyl ring. There are present strong (Desiraju & Steiner, 1999) intermolecular N—H···O hydrogen bonds (Tab. 1) that link the molecules into chains propagated in the direction [010].

### Experimental

Fresh benzaldehyde (6 mmol), ethyl acetoacetate (6 mmol) and NH_4_HCO_3_ (6 mmol) were  mixed in a 50 ml flask. After the mixture had been stirred for 3 h at 293 K, the crude product was obtained. The title crystals were obtained by recrystallization from ethanol, affording the title compound as a yellow block crystalline solid. Elemental analysis: calculated for C_19_H_23_NO_4_: C 69.28, H 7.04, N 4.25 weight%; found: C 69.29, H 7.85, N 4.29 weight%.

### Refinement

All the hydrogens were discernible in the difference electron density map. Except for the secondary-amine H atom whose coordinates were refined freely the remaining hydrogens were situated into the idealized positions and were refined within a riding model approximation: C_methyl_—H = 0.96, C_methylene_—H 0.97, C_methine_ = 0.98 Å. *U*_iso_(H) = 1.2 U_eq_C_methylene_/C_methine_/N_secondary-amine_; *U*_iso_(H) = 1.5 U_eq_(C_methyl_). The methyl groups were allowed to rotate during the refinement.

### Crystal data

**Table:**

C_19_H_23_NO_4_	*F*(000) = 704
*M**_r_* = 329.38	*D*_x_ = 1.250 Mg m^−^^3^
Monoclinic, *P*2_1_/*c*	Mo *K*α radiation, λ = 0.71073 Å
Hall symbol: -P2ybc	Cell parameters from 2342 reflections
*a* = 9.7502 (12) Å	θ = 2.6–27.7°
*b* = 7.3854 (9) Å	µ = 0.09 mm^−^^1^
*c* = 24.326 (2) Å	*T* = 298 K
β = 92.567 (1)°	Block, yellow
*V* = 1749.9 (3) Å^3^	0.50 × 0.46 × 0.32 mm
*Z* = 4	

### Data collection

**Table:**

Siemens SMART 1000 CCD diffractometer	3084 independent reflections
Radiation source: fine-focus sealed tube	1989 reflections with *I* > 2σ(*I*)
graphite	*R*_int_ = 0.033
φ and ω scans	θ_max_ = 25.0°, θ_min_ = 1.7°
Absorption correction: multi-scan (*SADABS*; Sheldrick, 1996)	*h* = −7→11
*T*_min_ = 0.958, *T*_max_ = 0.973	*k* = −8→8
8718 measured reflections	*l* = −28→28

### Refinement

**Table:**

Refinement on *F*^2^	Secondary atom site location: difference Fourier map
Least-squares matrix: full	Hydrogen site location: difference Fourier map
*R*[*F*^2^ > 2σ(*F*^2^)] = 0.045	H atoms treated by a mixture of independent and constrained refinement
*wR*(*F*^2^) = 0.131	*w* = 1/[σ^2^(*F*_o_^2^) + (0.0521*P*)^2^ + 0.8294*P*] where *P* = (*F*_o_^2^ + 2*F*_c_^2^)/3
*S* = 1.01	(Δ/σ)_max_ < 0.001
3084 reflections	Δρ_max_ = 0.22 e Å^−^^3^
225 parameters	Δρ_min_ = −0.18 e Å^−^^3^
0 restraints	Extinction correction: *SHELXL97* (Sheldrick, 2008), Fc^*^=kFc[1+0.001xFc^2^λ^3^/sin(2θ)]^-1/4^
85 constraints	Extinction coefficient: 0.0027 (8)
Primary atom site location: structure-invariant direct methods	

### Special details

**Table:**

Experimental. The sample was cut out from a larger slab crystal.
Geometry. All esds (except the esd in the dihedral angle between two l.s. planes) are estimated using the full covariance matrix. The cell esds are taken into account individually in the estimation of esds in distances, angles and torsion angles; correlations between esds in cell parameters are only used when they are defined by crystal symmetry. An approximate (isotropic) treatment of cell esds is used for estimating esds involving l.s. planes.
Refinement. Refinement of F^2^ against ALL reflections. The weighted R-factor wR and goodness of fit S are based on F^2^, conventional R-factors R are based on F, with F set to zero for negative F^2^. The threshold expression of F^2^ > 2sigma(F^2^) is used only for calculating R-factors(gt) etc. and is not relevant to the choice of reflections for refinement. R-factors based on F^2^ are statistically about twice as large as those based on F, and R- factors based on ALL data will be even larger.

### Fractional atomic coordinates and isotropic or equivalent isotropic displacement parameters (Å^2^)

**Table:**

	*x*	*y*	*z*	*U*_iso_*/*U*_eq_	
N1	0.3363 (2)	1.1267 (3)	0.55024 (8)	0.0405 (5)	
H1	0.327 (3)	1.230 (4)	0.5394 (10)	0.049*	
O1	0.57917 (16)	0.6975 (2)	0.65667 (7)	0.0476 (5)	
O2	0.66266 (19)	0.9781 (3)	0.66376 (8)	0.0619 (6)	
O3	0.14967 (17)	0.6965 (2)	0.46314 (6)	0.0468 (5)	
O4	0.2624 (2)	0.5071 (2)	0.51964 (7)	0.0572 (5)	
C1	0.4405 (2)	1.0939 (3)	0.58911 (9)	0.0376 (6)	
C2	0.4587 (2)	0.9233 (3)	0.60794 (9)	0.0332 (5)	
C3	0.3494 (2)	0.7821 (3)	0.59344 (8)	0.0324 (5)	
H3	0.3937	0.6630	0.5927	0.039*	
C4	0.2846 (2)	0.8200 (3)	0.53680 (8)	0.0318 (5)	
C5	0.2721 (2)	0.9937 (3)	0.51905 (9)	0.0356 (5)	
C6	0.2424 (2)	0.7790 (3)	0.63775 (8)	0.0323 (5)	
C7	0.2655 (2)	0.6791 (4)	0.68509 (10)	0.0469 (6)	
H7	0.3436	0.6070	0.6888	0.056*	
C8	0.1752 (3)	0.6838 (4)	0.72719 (10)	0.0565 (8)	
H8	0.1939	0.6175	0.7592	0.068*	
C9	0.0577 (3)	0.7862 (4)	0.72192 (10)	0.0538 (7)	
H9	−0.0035	0.7891	0.7501	0.065*	
C10	0.0315 (3)	0.8837 (4)	0.67494 (10)	0.0497 (7)	
H10	−0.0483	0.9524	0.6710	0.060*	
C11	0.1231 (2)	0.8805 (3)	0.63333 (10)	0.0411 (6)	
H11	0.1043	0.9481	0.6016	0.049*	
C12	0.5222 (3)	1.2590 (3)	0.60462 (11)	0.0547 (7)	
H12A	0.4892	1.3092	0.6379	0.082*	
H12B	0.5126	1.3469	0.5756	0.082*	
H12C	0.6172	1.2268	0.6102	0.082*	
C13	0.5757 (2)	0.8760 (3)	0.64511 (9)	0.0387 (6)	
C14	0.2330 (2)	0.6604 (3)	0.50671 (9)	0.0345 (5)	
C15	0.1963 (3)	1.0650 (3)	0.46890 (10)	0.0508 (7)	
H15A	0.2214	0.9965	0.4373	0.076*	
H15B	0.2196	1.1900	0.4638	0.076*	
H15C	0.0993	1.0543	0.4733	0.076*	
C16	0.6846 (3)	0.6355 (4)	0.69600 (11)	0.0582 (8)	
H16A	0.7690	0.7009	0.6902	0.070*	
H16B	0.7019	0.5079	0.6898	0.070*	
C17	0.6457 (4)	0.6618 (5)	0.75336 (12)	0.0819 (10)	
H17A	0.6362	0.7888	0.7606	0.123*	
H17B	0.7156	0.6118	0.7779	0.123*	
H17C	0.5601	0.6018	0.7589	0.123*	
C18	0.1024 (3)	0.5457 (3)	0.42944 (10)	0.0497 (7)	
H18A	0.0477	0.4640	0.4507	0.060*	
H18B	0.1800	0.4791	0.4162	0.060*	
C19	0.0187 (4)	0.6213 (4)	0.38256 (12)	0.0757 (10)	
H19A	−0.0562	0.6897	0.3962	0.114*	
H19B	−0.0167	0.5242	0.3598	0.114*	
H19C	0.0747	0.6988	0.3612	0.114*	

### Atomic displacement parameters (Å^2^)

**Table:**

	*U*^11^	*U*^22^	*U*^33^	*U*^12^	*U*^13^	*U*^23^
N1	0.0507 (13)	0.0230 (10)	0.0470 (12)	−0.0013 (10)	−0.0054 (10)	0.0019 (9)
O1	0.0410 (10)	0.0462 (11)	0.0542 (11)	0.0029 (8)	−0.0141 (8)	0.0051 (8)
O2	0.0573 (12)	0.0616 (12)	0.0647 (12)	−0.0180 (10)	−0.0213 (10)	0.0014 (10)
O3	0.0648 (11)	0.0324 (9)	0.0415 (9)	−0.0024 (8)	−0.0184 (8)	−0.0023 (7)
O4	0.0876 (14)	0.0259 (9)	0.0555 (11)	0.0040 (9)	−0.0245 (10)	0.0001 (8)
C1	0.0402 (14)	0.0340 (14)	0.0385 (13)	−0.0032 (11)	0.0009 (11)	−0.0053 (10)
C2	0.0344 (12)	0.0335 (13)	0.0316 (11)	−0.0016 (10)	0.0000 (10)	−0.0018 (10)
C3	0.0371 (13)	0.0252 (12)	0.0345 (12)	−0.0002 (10)	−0.0037 (10)	0.0014 (9)
C4	0.0373 (13)	0.0273 (12)	0.0305 (11)	0.0001 (10)	−0.0014 (10)	0.0010 (9)
C5	0.0425 (14)	0.0316 (12)	0.0327 (12)	0.0017 (11)	0.0009 (10)	0.0003 (10)
C6	0.0356 (13)	0.0285 (12)	0.0323 (12)	−0.0057 (10)	−0.0052 (9)	0.0015 (9)
C7	0.0388 (14)	0.0544 (17)	0.0468 (15)	−0.0019 (12)	−0.0055 (12)	0.0163 (12)
C8	0.0498 (16)	0.078 (2)	0.0414 (15)	−0.0118 (15)	−0.0034 (13)	0.0210 (14)
C9	0.0481 (16)	0.0715 (19)	0.0424 (15)	−0.0113 (15)	0.0079 (12)	0.0010 (14)
C10	0.0471 (15)	0.0519 (16)	0.0507 (16)	0.0061 (13)	0.0075 (13)	0.0032 (13)
C11	0.0452 (14)	0.0398 (14)	0.0382 (13)	0.0031 (12)	0.0006 (11)	0.0069 (11)
C12	0.0603 (17)	0.0389 (15)	0.0641 (17)	−0.0110 (13)	−0.0057 (14)	−0.0053 (13)
C13	0.0356 (13)	0.0459 (15)	0.0347 (13)	−0.0038 (12)	0.0019 (10)	−0.0027 (11)
C14	0.0421 (13)	0.0304 (13)	0.0309 (12)	−0.0003 (11)	−0.0005 (10)	0.0006 (10)
C15	0.0710 (18)	0.0355 (14)	0.0446 (15)	0.0015 (13)	−0.0099 (13)	0.0069 (11)
C16	0.0473 (16)	0.0625 (19)	0.0629 (18)	0.0087 (14)	−0.0179 (13)	0.0062 (14)
C17	0.091 (2)	0.098 (3)	0.0554 (19)	0.010 (2)	−0.0085 (17)	0.0202 (18)
C18	0.0689 (18)	0.0353 (14)	0.0436 (14)	−0.0102 (13)	−0.0112 (13)	−0.0056 (11)
C19	0.105 (3)	0.0559 (18)	0.0620 (19)	−0.0032 (18)	−0.0383 (18)	−0.0064 (15)

### Geometric parameters (Å, °)

**Table:**

N1—C5	1.375 (3)	C8—H8	0.9300
N1—C1	1.378 (3)	C9—C10	1.365 (4)
N1—H1	0.81 (3)	C9—H9	0.9300
O1—C13	1.348 (3)	C10—C11	1.380 (3)
O1—C16	1.447 (3)	C10—H10	0.9300
O2—C13	1.208 (3)	C11—H11	0.9300
O3—C14	1.333 (3)	C12—H12A	0.9600
O3—C18	1.445 (3)	C12—H12B	0.9600
O4—C14	1.206 (3)	C12—H12C	0.9600
C1—C2	1.349 (3)	C15—H15A	0.9600
C1—C12	1.495 (3)	C15—H15B	0.9600
C2—C13	1.466 (3)	C15—H15C	0.9600
C2—C3	1.522 (3)	C16—C17	1.475 (4)
C3—C4	1.516 (3)	C16—H16A	0.9700
C3—C6	1.533 (3)	C16—H16B	0.9700
C3—H3	0.9800	C17—H17A	0.9600
C4—C5	1.357 (3)	C17—H17B	0.9600
C4—C14	1.464 (3)	C17—H17C	0.9600
C5—C15	1.493 (3)	C18—C19	1.482 (4)
C6—C7	1.378 (3)	C18—H18A	0.9700
C6—C11	1.384 (3)	C18—H18B	0.9700
C7—C8	1.381 (3)	C19—H19A	0.9600
C7—H7	0.9300	C19—H19B	0.9600
C8—C9	1.374 (4)	C19—H19C	0.9600
			
C5—N1—C1	123.83 (19)	C1—C12—H12B	109.5
C5—N1—H1	116.7 (19)	H12A—C12—H12B	109.5
C1—N1—H1	117.3 (19)	C1—C12—H12C	109.5
C13—O1—C16	117.3 (2)	H12A—C12—H12C	109.5
C14—O3—C18	117.68 (17)	H12B—C12—H12C	109.5
C2—C1—N1	118.6 (2)	O2—C13—O1	121.4 (2)
C2—C1—C12	128.0 (2)	O2—C13—C2	126.6 (2)
N1—C1—C12	113.4 (2)	O1—C13—C2	111.9 (2)
C1—C2—C13	121.2 (2)	O4—C14—O3	121.6 (2)
C1—C2—C3	118.8 (2)	O4—C14—C4	123.6 (2)
C13—C2—C3	119.83 (19)	O3—C14—C4	114.84 (19)
C4—C3—C2	110.05 (17)	C5—C15—H15A	109.5
C4—C3—C6	111.88 (17)	C5—C15—H15B	109.5
C2—C3—C6	109.81 (17)	H15A—C15—H15B	109.5
C4—C3—H3	108.3	C5—C15—H15C	109.5
C2—C3—H3	108.3	H15A—C15—H15C	109.5
C6—C3—H3	108.3	H15B—C15—H15C	109.5
C5—C4—C14	125.3 (2)	O1—C16—C17	112.2 (2)
C5—C4—C3	119.46 (19)	O1—C16—H16A	109.2
C14—C4—C3	115.16 (18)	C17—C16—H16A	109.2
C4—C5—N1	117.9 (2)	O1—C16—H16B	109.2
C4—C5—C15	128.8 (2)	C17—C16—H16B	109.2
N1—C5—C15	113.3 (2)	H16A—C16—H16B	107.9
C7—C6—C11	117.4 (2)	C16—C17—H17A	109.5
C7—C6—C3	120.3 (2)	C16—C17—H17B	109.5
C11—C6—C3	122.23 (19)	H17A—C17—H17B	109.5
C6—C7—C8	121.4 (2)	C16—C17—H17C	109.5
C6—C7—H7	119.3	H17A—C17—H17C	109.5
C8—C7—H7	119.3	H17B—C17—H17C	109.5
C9—C8—C7	120.1 (2)	O3—C18—C19	107.3 (2)
C9—C8—H8	119.9	O3—C18—H18A	110.3
C7—C8—H8	119.9	C19—C18—H18A	110.3
C10—C9—C8	119.5 (2)	O3—C18—H18B	110.3
C10—C9—H9	120.2	C19—C18—H18B	110.3
C8—C9—H9	120.2	H18A—C18—H18B	108.5
C9—C10—C11	120.1 (2)	C18—C19—H19A	109.5
C9—C10—H10	119.9	C18—C19—H19B	109.5
C11—C10—H10	119.9	H19A—C19—H19B	109.5
C10—C11—C6	121.4 (2)	C18—C19—H19C	109.5
C10—C11—H11	119.3	H19A—C19—H19C	109.5
C6—C11—H11	119.3	H19B—C19—H19C	109.5
C1—C12—H12A	109.5		

### Hydrogen-bond geometry (Å, °)

**Table:**

*D*—H···*A*	*D*—H	H···*A*	*D*···*A*	*D*—H···*A*
N1—H1···O4^i^	0.81 (3)	2.19 (3)	2.986 (3)	168 (3)
C3—H3···O1	0.98	2.35	2.733 (3)	103
C3—H3···O4	0.98	2.43	2.816 (3)	103
C7—H7···O1	0.93	2.55	3.169 (3)	124
C12—H12C···O2	0.96	2.27	2.841 (3)	116
C15—H15A···O3	0.96	2.42	2.762 (3)	101
C8—H8···O2^ii^	0.93	2.51	3.387 (3)	157

Symmetry codes: (i) *x*, *y*+1, *z*; (ii) −*x*+1, *y*−1/2, −*z*+3/2.
